# Supplementary material for: Collateral sensitivity associated with antibiotic resistance plasmids
Source: eLife. 2021 Jan 20;10:e65130. doi: 10.7554/eLife.65130 (PMC7837676; doi:10.7554/eLife.65130)
Supplement: Supplementary file 1. — (A) ANOVA results of the growth curves (B), information on chromosomal mutations accumulated during plasmid acquisition (C), and the characteristics of the E. coli clinical isolates used in this study (D). [file elife-65130-supp1.docx]

**Supplementary File 1 for**

**Collateral Sensitivity Associated with Antibiotic Resistance Plasmids**

Cristina Herencias^1,a^, Jerónimo Rodríguez-Beltrán^1,2,a,^*, Ricardo León-Sampedro^1,2^, Aida Alonso-del Valle^1^, Jana Palkovičová^3^, Rafael Cantón^1,4^, Álvaro San Millán^1,2,5,^*.

^1^ *Servicio de Microbiología. Hospital Universitario Ramón y Cajal* and *Instituto Ramón y Cajal de Investigación Sanitaria*. Madrid, Spain.

^2^ *Centro de Investigación Biológica en Red* *Epidemiología y Salud Pública*, *Instituto de Salud Carlos III*. Madrid. Spain.

^3^ Department of Biology and Wildlife Diseases, Faculty of Veterinary Hygiene and Ecology, University of Veterinary and Pharmaceutical Sciences Brno, Czech Republic.

^4^ *Red Española de Investigación en Patología Infecciosa*. *Instituto de Salud Carlos III*. Madrid. Spain.

^5^ *Centro Nacional de Biotecnología-CSIC*, Madrid, Spain.

^a^ These authors contributed equally to this study and should be regarded as joint first authors.

* Correspondence: [asanmillan@cnb.csic.es](mailto:asanmillan@cnb.csic.es), [jeronimo.rodriguez.beltran@gmail.com](mailto:jeronimo.rodriguez.beltran@gmail.com)

## Supplementary file 1A. Antibiotic susceptibility results

|  | **Minimal inhibitory concentration (mg/L)** | | | | | | | | |
| --- | --- | --- | --- | --- | --- | --- | --- | --- | --- |
| **Drug class/target** | **Antibiotics** | MG1655 | | MG1655/  pOXA-48 | MG1655/  pKAZ3 | MG1655/  pKA2Q | MG1655/  pCF12 | MG1655/  pCEMR | MG1655/  pKP-M1144 |
|  |  | MH | LB |  |  |  |  |  |  |
| Β-lactam/  Cell wall synthesis | Amoxicillin-clavulanic acid (AMC) | 6 | 16 | >512 | 32 | 256 | 128 | 512 | 384 |
|  | Cefotaxime (CTX) | 0.03 | 0.375 | 8 | 8 | 8 | 8 | 8 | 8 |
|  | Ceftazidime (CAZ) | 0.5 | 0.375 | 0.5 | 32 | 32 | 32 | 32 | 32 |
|  | Ertapenem (ERT) | 0.01 | 0.062 | 0.187 | 0.507 | 0.125 | 0.125 | 0.25 | 0.75 |
| Aminoglycoside/ Protein synthesis (30S) | Gentamicin (GM) | 2 | 6 | 6 | 2 | 4 | 4 | 8 | 8 |
|  | Kanamycin  (KM) | 8 | 16 | 24 | 8 | 12 | 4 | 288 | 24 |
| Tetracycline/  Protein synthesis (30S) | Tetracycline (TET) | 2 | 2 | 4 | 32 | 1 | 1.5 | 4 | 4 |
|  | Tigecycline (TGC) | 0.5 | 1 | 1 | 1 | 1 | 1 | 1 | 1 |
| Amphenicol/  Protein synthesis (50S) | Chloramphenicol (CM) | 8 | 8 | 16 | 8 | 8 | 8 | 8 | 8 |
| Macrolide/  Protein synthesis (50S) | Azithromycin (AZI) | 16 | 16 | 8 | 12 | 12 | 8 | 12 | 12 |
| Quinolone/  DNA gyrase | Ciprofloxacin (CIP) | 0.5 | 0.5 | 0.75 | 8 | 1 | 8 | 2 | 3 |
| Rifamycin/  RNA polymerase | Rifampicin (RIF) | 8 | 8 | 8 | 24 | 24 | 8 | 16 | 256 |
| Polymyxin/ Lipopolysaccharide | Colistin (COL) | 4 | 8 | 4 | 6 | 8 | 6 | 8 | 6 |

MIC of the 13 antibiotics for *E.* coli MG1655 in LB and MH medium and the MIC of the six plasmid-carrying MG1655 strains in LB medium. Median value of 4-5 independent biological replicates.

## Supplementary file 1B. ANOVA results of the growth curves of plasmids producing CS effects

|  | **Area under the growth curve** | | |
| --- | --- | --- | --- |
| **Plasmid/Antibiotic** | **P-val** | **df** | **F** |
| pCF12/AZI | <0.000001 | 3 | 46.435 |
| pCF12/KM | 0.000008 | 3 | 8.859 |
| pKA2Q/TET | 0.460776 | 3 | 0.878 |
| pKAZ3/GM | 0.264279 | 3 | 1.368 |
| pKAZ3/KM | 0.025920 | 3 | 3.383 |
| pOXA-48/AZI | <0.000001 | 3 | 12.664 |
| pOXA-48/COL | <0.000001 | 3 | 5.9618 |

Results of the ‘plasmid x antibiotic concentration’ interaction term of ANOVA analyses for each plasmid-antibiotic combination.

## Supplementary file 1C. Chromosomal mutations accumulated during plasmid acquisition.

| **Strain** | **Gene** | **Gene product** | **Type** | **Position^A^** | **Reference^B^** | **Alternative** | **Effect** |
| --- | --- | --- | --- | --- | --- | --- | --- |
| MG1655/pKP-M1144 | *gadB* | Glutamate decarboxylase beta | SNP | 642/1401 | G | A | Synonymous (Thr214Thr) |
| MG1655/pKP-M1144 | *selB* | Selenocysteine-specific elongation factor | SNP | 1256/1845 | G | T | Missense (Trp419Leu) |
| MG1655/pCEMR | *ppk* | Polyphosphate kinase | SNP | 1488/2067 | T | C | Synonymous (Arg496Arg) |
| MG1655/pCEMR | *selB* | Selenocysteine-specific elongation factor | SNP | 1256/1845 | G | T | Missense (Trp419Leu) |
| MG1655/pKA2Q | *cpxA* | Sensor histidine kinase | SNP | 515/1374 | A | G | Missense (Met172Thr) |
| MG1655/pCF12 | Intergenic | - | Insertion | 1,212,080 | AGGGGGGGGT | AGGGGGGGGGT | - |

**^A^**Position relative to the start of the open reading frame, except for the intergenic mutation which refers to the chromosomal coordinate. **^B^**Reference genome is plasmid-free MG1655 (deposited in Sequence Read Archive [SRA], BioProject ID: PRJNA644278)

## Supplementary file 1D. Characteristics of the *E. coli* clinical isolates used in this study.

| **Strain** | **Phylogroup** | **Sequence Type** | **Plasmids** | **Resistance genes** |
| --- | --- | --- | --- | --- |
| Ec02 | B1 | 453 | Col-like, IncFIB, IncI, IncFIC, IncY | *aadA5, blaCTX-M-1, blaTEM-1B, dfrA17, sul2, tet(B), mdf(A)* |
| Ec03 | B2 | 131 | Col-like, IncFIB, IncFII, IncB/O/K/Z | *blaCTX-M-14, blaTEM-1B, mdf(A)* |
| Ec04 | B2 | 131 | Col-like, IncFIB, IncFII, IncFIA | *aadA5, aph(3'')-Ib, aph(6)-Id, blaCTX-M-14, blaTEM-1B, dfrA17, mph(A), sul1, sul2, tet(A), mdf(A)* |
| Ec06 | A | 10 | Col-like, IncFII, IncI | *aadA5, blaCTX-M-1, sul2, tet(A), mdf(A)* |
| Ec10 | D | 69 | Col-like, IncFII, pEC4115 | *aac(3)-Iva, aph(3'')-Ib, aph(4)-Ia, aph(6)-Id, blaCTX-M-1, mph(A), mdf(A)* |
| Ec18 | F | 648 | Col-like, IncY | *aadA5, blaCTX-M-14, dfrA17, mph(A), sul1, mdf(A)* |
| Ec20 | B2 | 131 | IncFIB, IncI, IncFIA, IncFIC | *aac(3)-Iid, blaCTX-M-15, blaTEM-1B, mdf(A)* |
| Ec21 | D | 405 | Col-like, IncFIB, IncFII, IncFIA, p0111 | *aac(3)-Iia, aph(3'')-Ib, aph(6)-Id, blaCTX-M-15, floR, sul2, tet(A), tet(B), mdf(A)* |
| Ec25 | A | 10 | IncFIB, IncFII, IncX, IncFIC | *aph(3')-Ia, aph(6)-Id, blaCTX-M-3, dfrA14, fosA3, sul2, mdf(A)* |
